# What Should I Trust? Individual Differences in Attitudes to Conflicting Information and Misinformation on COVID-19

**DOI:** 10.3389/fpsyg.2021.588478

**Published:** 2021-06-21

**Authors:** Petra Filkuková, Peter Ayton, Kim Rand, Johannes Langguth

**Affiliations:** ^1^Simula Research Laboratory, Oslo, Norway; ^2^Centre for Decision Research, University of Leeds, Leeds, United Kingdom; ^3^Health Services Research Centre, Akershus University Hospital, Akershus, Norway

**Keywords:** motivated reasoning, selective exposure, selective perception, evaluation of information, trust in misinformation, trust in authorities, precautionary behavior, COVID-19

## Abstract

The COVID-19 pandemic constitutes a novel threat and traditional and new media provide people with an abundance of information and misinformation on the topic. In the current study, we investigated who tends to trust what type of mis/information. The data were collected in Norway from a sample of 405 participants during the first wave of COVID-19 in April 2020. We focused on three kinds of belief: the belief that the threat is overrated (COVID-threat skepticism), the belief that the threat is underrated (COVID-threat belief) and belief in misinformation about COVID-19. We studied sociodemographic factors associated with these beliefs and the interplay between attitudes to COVID-19, media consumption and prevention behavior. All three types of belief were associated with distrust in information about COVID-19 provided by traditional media and distrust in the authorities' approach to the pandemic. COVID-threat skepticism was associated with male gender, reduced news consumption since the start of the pandemic and lower levels of precautionary measures. Belief that the COVID-19 threat is underrated was associated with younger age, left-wing political orientation, increased news consumption during the pandemic and increased precautionary behavior. Consistent with the assumptions of the theory of planned behavior, individual beliefs about the seriousness of the COVID-19 threat predicted the extent to which individual participants adopted precautionary health measures. Both COVID-threat skepticism and COVID-threat belief were associated with endorsement of misinformation on COVID-19. Participants who endorsed misinformation tended to: have lower levels of education; be male; show decreased news consumption; have high Internet use and high trust in information provided by social media. Additionally, they tended to endorse multiple misinformation stories simultaneously, even when they were mutually contradictory. The strongest predictor for low compliance with precautionary measures was endorsement of a belief that the COVID-19 threat is overrated which at the time of the data collection was held also by some experts and featured in traditional media. The findings stress the importance of consistency of communication in situations of a public health threat.

## Introduction

There is clear evidence that public reactions to health communications are influenced significantly by the characteristics of warning messages and that, in order to achieve optimal responses from the population, public health communications should have the attributes of specificity, consistency, certainty, clarity, accuracy and sufficiency (Mileti and Peek, 2000). Conflicting and confusing messages lead to misunderstandings and decreased credibility of the source, thereby reducing the efficacy of the communication (Nigg, 1987; Webster et al., 2020). Messages concerning the novel threat of COVID-19 have not been fully consistent over time, as authorities such as the World Health Organization (WHO) and countries' leaders have, in the light of emerging evidence, changed their evaluation of the seriousness of the disease, as well as their recommendations of measures to defend against it. An example of one controversy was the variation in messages about the use of face masks by asymptomatic individuals, which spanned from being discounted as a COVID-19 myth (McLaughlin, 2020), through warnings that risks associated with using face masks might outweigh their benefits (Lazzarino et al., 2020), to including them in official recommendations (BBC News, 2020; Brooks et al., 2020; Turak, 2020). Another topic of dispute was whether COVID-19 is airborne or whether close contact with an infected person is necessary for transmission to occur (Rabin and Anthes, 2021). Such inconsistent information can confuse the public, decrease trust in authorities and create anxiety about what information one should trust and which prevention measures to follow. Blurring of the line between fact and misinformation has also appeared in other COVID-19 news topics, for instance in relation to claims that COVID-19 originated from a research laboratory (Brewster, 2020; Wade, 2021).

People can also have various opinions about the COVID-19 threat in relation to the fact that they know that different countries have reached different conclusions regarding the gravity of the threat and required controlling measures. For instance Norway, the location of our study, introduced a national lockdown on 12th March 2020, whereas neighboring Sweden, which had at that point a similar number of infections, has resisted introducing a lockdown, despite steeply rising numbers of cases (COVID-19 Dashboard, CSSE—JHU, 2020^1^; Folkestad, 2020; Franks, 2020; Norrestad, 2020a,b). Even within the same country experts have expressed conflicting opinions and have sometimes criticized measures introduced in their own country (as in the case of our study Norway) as too strict (Berg Bentzrød and Dommerud, 2020; Mølsted, 2020) or too mild (Helljesen and Øverbø, 2020). Given the wide variety of expert opinions concering the seriousness of COVID-19 which were circulating in the media during the time of the data collection (i.e., the end of April 2020), it is to be expected that the lay population will also have varying attitudes and beliefs regarding the seriousness of the threat, as has been found for instance in the case of climate change beliefs (Heath and Gifford, 2006). Through the mechanism of motivated reasoning, people tend to seek out, pay attention to, remember, and trust information which corroborates their prior attitudes and discredits opposing information (Kahan, 2013). The mechanism of selective exposition, perception and retention has been found across various informational topic domains, including politics, climate change, and disease prevention (smoking cessation, HIV prevention) (e.g., Hwang, 2010; Flynn et al., 2017; Hartmann et al., 2018; Druckman and McGrath, 2019). Motivated reasoning helps to mitigate cognitive dissonance and earlier accepted misinformation is often retained even after one learns that it has been debunked (Nyhan and Reifler, 2015).

The requirement of clarity of communication is also a challenge, as news and public health communications on COVID-19 contain technical terms (such as basic reproduction number, exponential growth, fatality vs. mortality rate etc.). In common with some other threats, for instance radiation, the COVID-19 virus is invisible to the eye, which makes it harder for the public to fully appreciate and understand the danger, as opposed to, for instance, floods, tornadoes or fire (Mileti and Peek, 2000). The invisibility of the threat also provides more scope for individual evaluations and interpretations of the threat level.

As a result of any confusion in public health communications, people may turn to non-official channels for information. Social media contain an abundance of misinformation related to the pandemic, which some—including the World Health Organization—have referred to as the COVID-19 infodemic (Ali and Kurasawa, 2020; European Commission, 2020; WHO, 2020). The resort to such information channels can be further justified by the fact that some news items originally labeled as misinformation were later taken more seriously, such as the use of face masks in asymptomatic individuals or the possibility that the virus could have escaped from a laboratory mentioned above. Conspiracy theories tend to be associated with major events, epidemics, collective threats, and times of political instability (McHoskey, 1995; Grzesiak-Feldman, 2007; Douglas and Sutton, 2008; Sharp, 2008; Carey et al., 2020). Such events elicit aversive feelings of uncertainty and a lack of control, which, evidence suggests, motivates the development of conspiracy theories in order to help people to understand the situation and its causes and hence reduce uncertainty and confusion (Van Prooijen and Douglas, 2017). The COVID-19 pandemic constitutes a global threat involving many uncertainties and thus provides ideal conditions for the flourishing of conspiracy theories (Van Bavel et al., 2020). The search for easy explanations for pandemics has a long history: in medieval Europe, Jews were persecuted for being responsible for the plague (Brotherton, 2015) and more recently, in 1889/1890, the outbreak of the deadly Russian flu was associated with introduction of electricity (Knapp, 2020). In the current paper, we use the term COVID-19 misinformation for all types of false claims in relation to the pandemic, in line with the terminology of others, e.g. WHO in calling for actions to tackle misinformation on COVID-19 (World Health Organization, 2021).

The effect of misinformation on beliefs about the world is critical in the era of the Internet when misinformation spreads faster than ever before, as an ordinary person can, by one click, instantly share their text across the globe. The amount of misinformation on Internet platforms is on the rise (Lazer et al., 2018) and a recent study found that on social media (Twitter) false news spread even faster than true news (Vosoughi et al., 2018). Given that times of crisis engender the rise of conspiracy theories, the COVID-19 pandemic may further accelerate this trend. Here we investigate whether participants who have high trust in social media have different attitudes toward the pandemic than participants who trust traditional media (TV, radio, printed newspapers). Traditional media, in contrast to many Internet sources, typically adhere to journalistic practices, ethical codices and content is subject to review and approval (e.g., by editors) prior to publication to the broad public. Their information quality is hence expected to be higher in comparison with information on social media platforms, particularly in countries where the media content is not subject to state censorship or control. According to the 2020 World Press Freedom Index, Norway has been evaluated as the country with the highest degree of freedom of speech (Reporters without Borders, 2020) and hence the information content of Norwegian television, radio, and newspapers is expected to be superior to information which one can find on social media.

The theory of planned behavior (TPB) links beliefs to behavior and has mainly been applied in research on behaviors related to protection of health and environment (Ajzen, 1991; Armitage and Conner, 2001; Xu et al., 2020). Although TPB has been criticized (Sniehotta et al., 2014), it is still in use and for instance recently proved successful in predicting participants' willingness to self-isolate during a hypothetical pandemic in China (data collected before the outbreak of COVID-19) (Zhang et al., 2020). Our study investigates the impact of beliefs on precautionary health behavior, while also simultaneously exploring the impact of media on one's beliefs about COVID-19.

The present study was conducted in Norway in April 2020 when many attributes of the new virus were still unknown, predictions of the development of the pandemic were unclear and the topic of the COVID-19 pandemic was prominent in news headlines. The first person infected with COVID-19 in Norway was identified on February 26th and on March 12th Norway introduced strict measures, including travel restrictions and the closing of educational institutions and sport facilities. At the time of the data collection in late April, some of the measures had already been lifted and the Norwegian government had promoted installation of the tracking app Smittestopp to help in preventing the disease spread.

The aim of our study is to explore factors associated with three types of beliefs related to COVID-19:

1. Belief that the threat of COVID-19 is serious and underrated (“COVID-threat belief”)
2. Belief that the threat of COVID-19 is mild and the situation is overrated (“COVID-threat skepticism”)
3. Belief in misinformation on COVID-19

Specifically, we will address three questions:

1. Which sociodemographic factors are associated with these beliefs?
2. How are media exposure and trust in media and the authorities associated with these beliefs?
3. How do these beliefs affect reported precautionary behavior?

## Method

### Participants

A total of 405 participants (48.4% men, 51.6% women) living in Norway participated in the study, the mean age of the sample was 48.1 years (age range 18–85 years). The participants were recruited from a representative panel of the Norwegian population (≥18 years of age) owned by Polling & Statistics AS, which was entrusted to send out the questionnaire. The participants from the panel filled out the online survey in the period 24–27th April 2020. The data was automatically stored in an SPSS file, Polling & Statistics AS subsequently rewarded the participants in the same way as in other data collections managed by the company.

### Measures

Participants were asked about sociodemographic variables of age, gender, level of education, marital status, employment status at the start of the pandemic, migration background, number of persons living in their household, and whether they lived in a rural or urban area. Participants rated their political orientation on an 11-point Likert scale (0 = left, 10 = right).

In order to investigate the effect of beliefs about COVID-19 on behavior (presumed by TPB), participants evaluated a list of statements expressing different beliefs about COVID-19 and reported the extent to which they complied with the precautionary measures. Participants rated on four-point scales their level of agreement (1 = fully disagree, 4 = fully agree) with a set of statements related to the COVID-19 pandemic and which appeared in the media in the period prior to the data collection (see Supplementary Material for the English version of the items which were presented in Norwegian). Seven statements emphasized the severity of COVID-19 (e.g., “people who were infected with COVID-19 will experience long-term negative health impacts,” “the COVID-19 pandemic is still at the start and many more people will die by the end of the year”) and their mean score identified “COVID-threat belief.” Eight statements downplayed the severity of the disease (e.g., “Norway overreacted and the measures against COVID-19 were too strict,” “the COVID-19 pandemic is almost over”) the mean score for which was identified as “COVID-threat skepticism.”

The full list of statements rated by participants also included eight examples of misinformation on COVID-19 being spread on the Internet (e.g., “consumption of the Corona brand of beer has an effect on the spread of COVID-19,” “the 5G network has an effect on the spread of COVID-19”). These statements were evaluated as misinformation at the time of the data collection and at the time of writing this paper this remained unchanged. However, we cannot exclude the (unlikely) possibility that their evaluation may change in the future, as sometimes misinformation (rumors, conspiracy theories etc.) turn out to be true (Flynn et al., 2017). We again computed a mean score for these eight items, which we further refer to as “trust in misinformation.” Additionally, eight statements expressed trust in authorities in relation to the pandemic (e.g., “the Norwegian Institute of Public Health has handled the pandemic correctly,” “the World Health Organization has handled the pandemic correctly”).

On a three-point scale (0 = no, 1 = sometimes, 2 = yes), participants rated how much they followed each of the eleven listed health measures (e.g., avoiding physical contact, frequently washing hands).

Participants were also asked about the estimated weekly number of hours they spend following news in total (TV, radio, newspaper, Internet) and the number of hours spent using the Internet (excluding watching movies online and playing online games, as we were primarily interested in hours of Internet use in which COVID-19 related content could have been encountered). Additionally, they were asked to evaluate on a five-point scale whether they reduced or increased their news consumption compared to the period before the pandemic (1 = reduced a lot, 5 = increased a lot). Participants further rated how much they trusted information on COVID-19 from different types of media: TV, radio, printed newspapers, and social media (1 = don't trust it at all, 5 = completely trust). Participants also evaluated how difficult they find it to distinguish facts from misinformation on the Internet (1 = very difficult, 5 = very easy). Several additional measures were taken, not reported in this paper.

The questionnaire items were constructed to be relevant and specific for the situation in Norway in April 2020 and reflected the status of knowledge and opinions about COVID-19 which appeared in media in that period, as well as the then health recommendations and misinformation. At the time of the data collection, the number of new cases in Norway was in decline and precautionary measures started to be lifted. As the threat was still novel and knowledge about COVID-19 was limited, it was a matter of opinion whether the precautionary measures should be evaluated as too strict or too mild, as well as whether the COVID-19 pandemic was perceived as close to the end or still at the start. In April 2020 it was not clear that the pandemic would be long-lasting and thus the measures of attitudes were not constructed with the aim of being universally applicable for all countries and all stages of the pandemic. The differences in attitudes toward COVID-19 likely did not cease to exist, yet in potential future data collections the questionnaire items would need to be modified to reflect changes in the development of the pandemic and knowledge about it.

### Analyses

Responses were combined into scales: COVID-threat belief (seven items), COVID-threat skepticism (eight items), misinformation belief (eight items), trust in authority (eight items), precautionary health behavior (eleven items), and trust in traditional media (TV, radio, printed newspapers).

Associations between these scales and other variables of interest (media consumption, trust in media, age, political orientation) were computed using Pearson's correlation. Regression models were developed to investigate which factors predicted COVID-threat belief, COVID-threat skepticism, misinformation belief, and precautionary health behavior. Data were analyzed using IBM SPSS Statistics 27 and R version 3.5.1.

## Results

Before investigating the association between variables, we provide a brief overview of the overall attitudes of the sample. For the entire sample we observe that on a 4-point scale, COVID-threat belief reached a higher mean score (*M* = 2.30, *SD* = 0.50) than COVID-threat skepticism (*M* = 1.54, *SD* = 0.45); *t*(404) = 20.76, *p* < 0.001, *partial* η^2^ = 0.516. The sample had an overall high trust in authorities in handling the pandemic (*M* = 3.09, *SD* = 0.50) and distrusted misinformation on COVID-19 (*M* = 1.21, *SD* = 0.27). Participants indicated that they trusted information about COVID-19 from traditional media (*M* = 3.70, *SD* = 0.74) more than from social media (*M* = 2.50, *SD* = 1.13); *t*(404) = 20.68, *p* < 0.001, *partial* η^2^ = 0.514.

In comparison with men, women were less skeptical about the threat of COVID-19 [*t*(359.12) = 4.66, *p* < 0.001], increased their reported news consumption more during the pandemic [*t*(403) = −2.84, *p* = 0.005], trusted misinformation less [*t*(402) = 2.16, *p* = 0.031] and followed the recommended health measures more [*t*(381.15) = −4.90, *p* < 0.001] (see Table 1).

**Table 1:** Gender comparison of attitudes toward COVID-19 and use of and trust in media (Independent samples *t*-test).

	**Male (*N* = 196) *M (SD)***	**Female (*N* = 209) *M (SD)***	***df***	***T***	***p***	***Partial η^2^***
COVID-threat belief^a^	2.27 (0.48)	2.34 (0.52)	403	−1.49	0.137	0.005
COVID-threat skepticism^a^	1.65 (0.50)	1.44 (0.37)	359.12	4.66	<0.001	0.052
Trust in misinformation^a^	1.24 (0.29)	1.18 (0.24)	402	2.16	0.031	0.011
Trust in authorities^a^	3.05 (0.54)	3.14 (0.46)	386.16	−1.80	0.073	0.008
Health measures^b^	1.33 (0.29)	1.46 (0.25)	381.15	−4.90	<0.001	0.057
Weekly hours of news consumption (traditional media, Internet)	10.80 (11.72)	10.48 (10.37)	403	0.289	0.772	<0.001
Increase in news consumption during the pandemic^c^	3.84 (0.91)	4.11 (0.95)	403	−2.84	0.005	0.020
Weekly hours of Internet use	20.10 (52.68)	15.77 (14.14)	403	1.14	0.253	0.003
Trust in information about COVID-19 from traditional media^c^	3.65 (0.83)	3.74 (0.66)	403	−1.24	0.217	0.004
Trust in information about COVID-19 from social media^c^	2.45 (1.17)	2.54 (1.08)	403	−0.82	0.413	0.002

a *Mean of scales rated 1–4*.

b *Mean of scales rated 0–2*.

c *Scales rated 1–5*.

A 2 x 2 ANOVA with gender and cohabiting status during the pandemic (living alone vs. with somebody) as between-subjects factors revealed the main effects of gender [*F*_(1, 401)_ = 33.12, *p* < 0.001, *partial* η^2^ = 0.076] and cohabiting status [*F*_(1, 401)_ = 6.51, *p* = 0.011, *partial* η^2^ = 0.016] on adherence to precautionary measures. Participants who lived alone tended to follow health measures less (*M* = 1.34, *SD* = 0.33) than participants living with somebody (*M* = 1.42, *SD* = 0.26). There was an interaction effect between gender and living alone [*F*_(1, 401)_ = 8.14, *p* = 0.005, *partial* η^2^= 0.020]: whereas women followed health measures equally regardless whether they were living alone (*M* = 1.47, *SD* = 0.26) or with somebody (*M* = 1.46, *SD* = 0.24) during the pandemic, men followed health measures significantly less when they lived alone (*M* = 1.19, *SD* = 0.34 vs. *M* = 1.37, *SD* = 0.27). This is in line with the finding that marital status had an impact on following the health measures [*F*_(3, 401)_ = 4.79, *p* < 0.001, *partial* η^2^= 0.034], with single participants following health measures the least (*M* = 1.26, *SD* = 0.32) and married participants following measures the most (*M* = 1.42, *SD* = 0.26). The variables living alone and marital status did not have a significant effect on any other of the investigated variables. Type of settlement (urban vs. rural) did not have any significant effect on any of the investigated variables.

### Associations Between Beliefs About COVID-19, Precautionary Behavior, and Media Consumption

We observed a significant relationship between beliefs about the level of seriousness of COVID-19 threat and prevention behavior, see Table 2. Participants who believed that COVID-19 is a very serious threat followed health measures more (*r* = 0.230, *p* < 0.001), whereas participants who were skeptical toward the COVID-19 threat reported less prevention behavior (*r* = −0.383, *p* < 0.001). Beliefs about the level of seriousness of the COVID-19 threat were also associated with changes in following news: whereas participants who believed that COVID-19 is a serious and underrated threat tended to increase their news consumption after the start of the pandemic (*r* = 0.155, *p* = 0.002), participants who were skeptical toward the COVID-19 threat decreased their exposure to news when the pandemic started (*r* = −0.227, *p* < 0.001). Participants with more extreme views on the level of the COVID-19 threat (both in the direction of underestimation and overestimation) tended to be younger and distrust authorities in handling the pandemic, whereas older participants tended to trust authorities in managing the situation. More extreme views on the evaluation of the threat were also associated with trust in misinformation related to COVID-19, the association was particularly strong for participants who were more skeptical about the seriousness of the threat of COVID-19 (*r* = 0.425, *p* < 0.001). Participants who tended to believe that the threat of COVID-19 is underestimated tended to be more left-wing politically (left-right scale: *r* = −0.167, *p* = 0.001).

**Table 2:** Associations between beliefs about COVID-19, precautionary behavior and media consumption.

	**COVID-threat belief**	**COVID-threat skepticism**	**Trust in misinformation**	**Trust in authorities**	**Health measures**	**Hours of news consumption**	**Change in news consumption (decrease-increase)**	**Hours of Internet use**	**Distinguishing facts from misinformation**	**Trust traditional media**	**Trust social media**	**Political orientation (left-right)**	**Age**
COVID-threat belief	-	−0.207^**^	0.108^*^	−0.340^**^	0.230^**^	0.101^*^	0.155^**^	0.071	−0.083	−0.121^*^	0.070	−0.167^**^	−0.220^**^
COVID-threat skepticism		-	0.425^**^	−0.398^**^	−0.383^**^	−0.019	−0.227^**^	0.089	−0.166^**^	−0.290^**^	−0.094	0.086	−0.130^**^
Trust in misinformation			-	−0.341^**^	−0.087	0.018	−0.153^**^	0.204^**^	−0.216^**^	−0.328^**^	0.104^*^	0.090	−0.094
Trust in authorities				-	0.112^*^	0.029	0.123^*^	−0.080	0.239^**^	0.486^**^	0.103^*^	−0.023	0.202^**^
Health measures					-	0.113^*^	0.200^**^	0.057	−0.100^*^	0.074	−0.035	−0.050	0.066
Hours of news consumption						-	0.220^**^	0.436^**^	0.080	0.083	0.037	−0.098^*^	0.086
Increase in news consumption							-	−0.019	0.047	0.203^**^	0.048	−0.038	0.134^**^
Hours of Internet use								-	−0.037	0.039	0.077	−0.070	−0.095
Distinguishing facts from misinformation									-	0.368^**^	0.011	−0.140^**^	−0.144^**^
Trust traditional media										-	0.273^**^	−0.160^**^	0.083
Trust social media											-	−0.087	0.115^*^
Political orientation (left-right)												-	−0.060
Age													-

* *p ≤ 0.05*;

** *p ≤ 0.001*.

Rated trust in misinformation was positively correlated with the amount of Internet use (*r* = 0.204, *p* < 0.001) and negatively associated with trust in the authorities handling the pandemic (*r* = −0.341, *p* < 0.001). Interestingly, trust in misinformation did not have any significant effect on precautionary behavior (*r* = −0.087, *p* = 0.081). However, a more detailed analysis revealed that two specific misinformation items (both concerning vaccination) were weakly negatively correlated with precautionary behavior: “Vaccine against COVID-19 will be available by summer” (*r* = −0.116, *p* = 0.022) and “The magnitude of COVID-19 is exaggerated in order to persuade the world's population to take a vaccine” (*r* = −0.133, *p* = 0.008) (see **Table 4**). Participants who trusted misinformation more tended to distrust traditional media in covering the pandemic (*r* = −0.328, *p* < 0.001) and instead trusted social media (*r* = 0.104, *p* = 0.037). At the same time, participants endorsing COVID-19 misinformation reported experiencing difficulty with distinguishing facts from misinformation (*r* = −0.216, *p* < 0.001).

By contrast, participants who trusted information on COVID-19 provided by traditional media simultaneously tended to trust the authorities handling the pandemic (*r* = 0.486, *p* < 0.001), tended to be more left-wing politically (left-right scale: *r* = −0.160, *p* = 0.001) and felt that it was rather easy to distinguish facts from misinformation (*r* = 0.368, *p* < 0.001). Level of trust in information on the pandemic from traditional media was not significantly associated with precautionary behavior (*r* = 0.074, *p* = 0.137), whereas trust in authorities handling the pandemic was positively associated with adoption of the precautionary health measures (*r* = 0.112, *p* = 0.024).

The regression model predicting COVID-threat belief identified none of the sociodemographic variables included in the model as significant predictors, while the increase in news consumption compared to the period before the pandemic was a significant predictor (*p* <0.001) (see Table 3). COVID-threat skepticism was predicted by lower age (*p* = 0.029), male gender (*p* = 0.029) and decreased news consumption during the pandemic (*p* < 0.001). Having achieved at least Masters level of education was a statistically significant predictor of greater trust in traditional media (*p* = 0.039), as was increased news consumption during the pandemic (*p* < 0.001), trust in social media (*p* < 0.001), lower reported COVID-threat belief (*p* < 0.001), and lower reported COVID-threat skepticism (*p* < 0.001).

**Table 3:** Linear models.

**Dependent variable**	**COVID-threat belief**				**COVID-threat skepticism**				**Trust in traditional media**				**Trust in misinformation**				**Precautionary behavior**			
**Predictors**	**Coef**.	**(*SE*)**	***Beta***	***p***	**Coef**.	**(*SE*)**	***Beta***	***p***	**Coef**.	**(*SE*)**	***Beta***	***p***	**Coef**.	**(*SE*)**	***Beta***	***p***	**Coef**.	**(*SE*)**	***Beta***	***p***
(Intercept)	2.155	(0.185)		<0.001	2.156	(0.167)		<0.001	4.193	(0.406)		<0.001	0.527	(0.144)		<0.001	1.244	(0.166)		< .001
Age (years)	−0.004	(0.002)	−0.213	0.058	−0.004	(0.002)	−0.108^*^	0.029	−0.001	(0.003)	−0.058	00.664	0.001	(0.001)	−0.011	0.628	0.002	(0.001)	0.067	0.175
Sex (female)	0.296	(0.153)	0.061	0.053	−0.303	(0.139)	−0.213^*^	0.029	0.045	(0.214)	−0.055	0.834	0.068	(0.076)	0.006	0.371	0.110	(0.080)	0.139	0.167
Age^*^sex interaction	−0.005	(0.003)	−0.08	0.102	0.002	(0.003)	0.041	0.395	−0.003	(0.004)	−0.029	0.529	−0.001	(0.001)	−0.04	0.361	−0.001	(0.002)	−0.02	0.654
High school education	−0.065	(0.127)	−0.061	0.608	0.100	(0.115)	0.103	0.382	0.202	(0.175)	00.128	0.248	−0.066	(0.062)	−0.113	0.289	−0.011	(0.066)	−0.019	0.862
Bachelor education	−0.170	(0.127)	−0.162	0.180	0.109	(0.115)	0.113	0.344	0.249	(0.176)	00.16	0.157	−0.122	(0.062)	−0.212	0.051	0.051	(0.066)	0.086	0.443
Master education	−0.177	(0.127)	−0.166	0.165	0.076	(0.115)	0.079	0.507	0.365	(0.176)	0.231^*^	0.039	−0.191	(0.063)	−0.327^**^	0.002	0.039	(0.067)	0.066	0.555
Change in news consumption (decrease-increase)	0.106	(0.026)	0.202^***^	<0.001	−0.085	(0.023)	−0.176^***^	<0.001	0.132	(0.037)	0.169^***^	<0.001	−0.020	(0.013)	−0.071	0.122	0.023	(0.014)	0.078	0.103
Trust in social media	0.025	(0.021)	0.057	0.241	−0.026	(0.019)	−0.065	0.177	0.160	(0.030)	0.245^***^	<0.001	0.037	(0.011)	0.151^***^	0.001	−0.026	(0.012)	−0.108^*^	0.025
Precautionary behavior									−0.019	(0.135)	−0.007	0.887	0.100	(0.048)	0.102^*^	0.038				
COVID-threat skepticism									−0.515	(0.083)	−0.317^***^	<0.001	0.293	(0.030)	0.487^***^	<0.001	−0.203	(0.034)	−0.332^***^	<0.001
COVID-threat belief									−0.319	(0.073)	−0.215^***^	<0.001	0.070	(0.026)	0.128^**^	0.008	0.100	(0.028)	0.179^***^	<0.001
Trust in misinformation																	0.114	(0.054)	0.112^*^	0.036
Trust in traditional media																	0.007	(0.019)	0.018	0.735

*Coef., unstandarized coefficient; Beta, standardized coefficient; SE, standard error*.

* *p ≤ 0.05*;

** *p ≤ 0.01*;

*** *p ≤ 0.001*.

Precautionary behavior was not predicted by any of the sociodemographic data but decreased with trust in social media (*p* = 0.025) and COVID-threat skepticism (*p* > 0.001) and increased with COVID-threat belief (*p* < 0.001) and belief in misinformation (*p* = 0.036). Interestingly, among those participants who were skeptical of COVID-threat, trust in misinformation was associated with more precautionary behavior. Thus, trust in misinformation turned out to be a predictor of precautionary behavior, since it motivated COVID-threat skeptics to compliance with precautionary measures.

### Belief in Misinformation on COVID-19

Recall that we measured the extent to which respondents indicated trust in misinformation using eight statements that expressed false information about the pandemic. Using Pearson's correlation coefficient, we observe that participants who trusted one type of misinformation on COVID-19 were also more likely to trust other misinformation messages (see Table 4). Some of these combinations could be a part of one narrative, for instance that the 5G network is responsible for the spread of COVID-19 and that the effect of COVID-19 is overblown so that everyone will take a vaccine (*r* = 0.412, *p* < 0.001). On social media these two types of misinformation sometimes appear in a narrative that claims that, after taking the vaccine, 5G masts will be able to start mind-controlling people (Radio Free Europe/Radio Liberty (RFL/RL), 2020); trust in multiple misinformation stories simultaneously could explain how such syntheses arise. However, in other cases, there was a positive association between misinformation messages which seem to oppose each other (e.g., 5G masts are responsible for the spread of COVID-19 and refugees are responsible for the spread of COVID-19, *r* = 0.208, *p* < 0.001).

**Table 4:** Correlations between trust in separate misinformation items and precautionary health behavior.

	**5G causes COVID-19**	**COVID-19 is used to persuade people to take a vaccine**	**Vaccine will be ready by summer**	**Refugees are responsible for the pandemic**	**Garlic is a remedy against COVID-19**	**Mosquitoes spread COVID-19**	**Corona beer causes COVID-19**	**COVID-19 was first outside China**	**Precautionary behavior**
5G causes COVID-19	-	0.412^**^	0.176^**^	0.208^**^	0.159^**^	0.153^**^	0.207^**^	0.094	−0.052
COVID-19 is used to persuade people to take a vaccine		-	0.168^**^	0.240^**^	0.221^**^	0.150^**^	0.096	0.105^*^	−0.133^**^
Vaccine will be ready by summer			-	0.179^**^	0.127^*^	0.096	0.116^*^	0.155^**^	−0.116^*^
Refugees are responsible for the pandemic				-	−0.005	0.161^**^	0.026	0.100	0.042
Garlic is a remedy against COVID-19					-	0.112^*^	0.169^**^	0.093	−0.064
Mosquitoes spread COVID-19						-	0.063	0.096	0.076
Corona beer causes COVID-19							-	0.060	−0.029
COVID-19 was first outside China								-	−0.027

* *p ≤ 0.05*;

** *p ≤ 0.001*.

**Figure 1:**
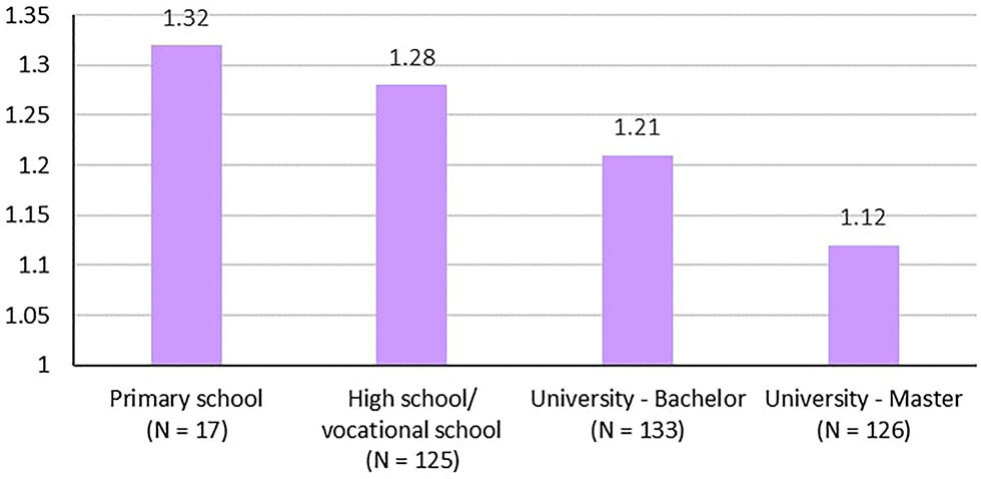
Highest achieved education and trust in misinformation.

When investigating sociodemographic variables associated with trust in misinformation on COVID-19, we observe that, in addition to male gender mentioned above, the level of education also had an effect and participants with higher achieved education were less likely to believe misinformation stories; *F*_(3, 396)_ = 7.97, *p* < 0.001, *partial* η^2^ = 0.057 (see Figure 1). The other investigated sociodemographic variables such as living alone, civic status and type of settlement did not have any association with trust in misinformation.

As reflected in Table 3, trust in misinformation was lower with higher level of education (*p* = 0.002), but increased with trust in social media (*p* = 0.001), with more precautionary behavior (*p* = 0.038), and with both higher reported COVID-threat belief (*p* = 0.008) and COVID-threat skepticism (*p* < 0.001).

## Discussion

This study shows that there are intelligible relationships between people's beliefs and doubts about the seriousness of the pandemic, their trust in authorities, their susceptibility to misinformation and their engagement with precautionary behaviors. The findings reveal that one's beliefs about the seriousness of the COVID-19 threat predict the extent to which individuals adopt precautionary health measures. Whereas COVID-threat believers applied many precautionary measures, COVID-threat skepticism was associated with decreased precautionary behavior. The congruence between beliefs and behavior is consistent with the ideas behind the theory of planned behavior (Ajzen, 1991). The mechanism of motivated reasoning and selective exposure can explain why COVID-threat believers increased their news consumption since the start of the pandemic, whereas COVID-threat skeptics decreased it. In other studies, high levels of news consumption in times of crises (e.g., terrorist attacks, COVID-19 pandemic) were associated with higher levels of anxiety and other psychological symptoms, which would be congruent with the association between increased news consumption and heightened worry about the seriousness of the threat (e.g., Ahern et al., 2002; Schlenger et al., 2002; Nekliudov et al., 2020). The causality may be bidirectional, as worried individuals may seek out information which resonates with their beliefs and being exposed to such news can even further increase their threat appraisal of the situation. Evaluation of the COVID-19 threat as very serious and underrated was further correlated with left-wing orientation; skepticism toward the COVID-19 threat was associated with male gender and reported difficulties in distinguishing facts from misinformation. Both of these extreme views of the threat (in the direction of underestimation and overestimation) were associated with younger age, distrust in authorities in handling the pandemic, distrust in information on COVID-19 provided by traditional media and tendency to endorse COVID-19 misinformation.

Norway is among those countries with the highest trust in authorities in the world (Ortiz-Ospina and Roser, 2016) and hence it is not surprising that the sample exhibited an overall high trust in authorities in handling the COVID-19 pandemic. This trust could have been further bolstered by the fact that, at the time of the data collection in the second half of April, measures introduced in mid-March had already shown an effect and COVID-19 was receding in Norway. According to the study by Rieger and Wang (2020) conducted shortly before our data collection, Norway had the fourth highest trust in the government in handling the pandemic out of 57 investigated countries and was preceded only by Vietnam, Quatar, and New Zealand. Simultaneously, Rieger and Wang (2020) found that countries with a higher trust in the government had a lower COVID-19 death toll. In our study, trust in authorities was positively associated with higher age, trust in media, and the amount of adopted prevention measures and negatively associated with trust in COVID-19 misinformation and with beliefs that the threat of COVID-19 is either underestimated or overestimated. The association between trust in authority and compliance with rules was also found in a study on tax payments and was explained by perception of fairness (Murphy, 2004). Murphy discusses the possibility that trust may be more efficient than punishment in promoting rules, which could plausibly also apply to the pandemic situation. Norway took the pandemic seriously and acted with caution, held the borders closed longer than the EU countries and, simultaneously, the trust of the population in the authorities and traditional media was high. Possibly in relation to that, the basic reproduction number for COVID-19 decreased to under 1 within about 2 weeks after the introduction of the lockdown and the country got the pandemic quickly under control (Franks, 2020). It would be interesting to investigate people's beliefs about COVID-19 in countries where the level of trust in authorities is low and/or where the severity of the threat was downplayed by the authorities.

Perhaps due to the generally high level of trust in authorities in Norway, trust in misinformation was not prevalent in our sample. Van Bavel et al. (2020) stressed the importance of fighting misinformation during the pandemic, however, the level of trust in misinformation can differ across countries. In our study, trust in misinformation was positively associated with male gender, lower education, high amount of Internet use, perception that the threat either overestimated or underestimated, trust in information on COVID-19 from social media and reported difficulty in distinguishing facts from misinformation. However, in a linear regression the effect of gender was not significant. This is at variance with the finding by Pennycook and Rand (2019) that men are better than women at differentiating facts from misinformation. People endorsing misinformation on COVID-19 tended to distrust information on the pandemic provided by traditional media (TV, radio, printed newspapers) and to decrease their news consumption after the start of the pandemic. Avoidance, or discounting, of information inconsistent with one's beliefs can be again explained by the protective mechanism of motivated reasoning. In contrast to studies by Pennycook and Rand (2019) conducted in the United States, we did not find any association between political orientation and trust in misinformation.

We found that trust in one type of misinformation was positively correlated with trust in other misinformation stories, even in cases when they provided contradictory explanations for the pandemic. Previous studies (e.g., Swami et al., 2010; Wood et al., 2012) have shown that those who believe in one conspiracy theory are also likely to endorse other conspiracy theories even in cases when they are mutually exclusive. Our study suggests that this finding extends to other types of misinformation. Simultaneous trust in different misinformation stories simultaneously possibly explains how they could blend together—in order to reduce cognitive dissonance from trusting seemingly opposing stories (e.g., “5G masts are responsible for the spread of COVID-19” and “the threat of COVID-19 is exaggerated so that everyone would take a vaccine”), new stories containing elements of the original stories could arise (after everybody takes the vaccine, people will be mind-controlled by 5G masts) (Radio Free Europe/Radio Liberty (RFL/RL), 2020). However, more research is needed to understand the exact mechanism underlying the creation of new misinformation narratives, as well as to address the problem as to why certain segments of the population distrust and avoid official news sources and instead turn to social media and misinformation.

One unexpected finding from our study was that there was no significant correlation between trust in misinformation on COVID-19 and precautionary behavior. A finer grained analysis revealed that the direction of association between adoption of precautionary measures and trust in misinformation is contingent upon the content of the specific misinformation item. The absence of a significant association between COVID-19 conspiracy beliefs and compliance with preventive measures was also found in a study conducted in Turkey (Alper et al., 2020). Yet another study discovered that belief in conspiracy theories was predicted by stressful life events and greater perceived stress (Swami et al., 2016). High levels of stress could possibly explain why people endorsing conspiracy theories are not necessarily relaxed about precautionary measures. In fact, linear regression revealed that participants who endorsed COVID-threat skepticism and simultaneously trusted misinformation items complied more with precautionary measures than participants who only endorsed COVID-threat skepticism.

Scientists, as well as international organizations, have called for measures against COVID-19 misinformation (European Commission, 2020; Van Bavel et al., 2020) and social media platforms have made significant efforts to remove misinformation related to COVID-19 from their websites (Guynn, 2020). In this context our failure to observe a negative association between belief in misinformation about COVID-19 and precautionary behavior is striking. We suspect that this finding may be related to the heterogeneity of misinformation stories. Whereas the belief that dramatic photos showing COVID-19 casualties are staged can certainly negatively impact one's precautionary behavior, it is unlikely that the belief that COVID-19 was manufactured in a Russian laboratory will have the same impact on one's risk perception.

Based on our findings, decreased precautionary behavior is strongly associated with attitudes downplaying the seriousness of the virus. The impact of beliefs and doubts about severity of a disease on compliance with precautionary measures is consistent with the assumptions of the theory of planned behavior (Ajzen, 1991) and, for instance, was also found during the 2009 H1N1 pandemic (Rubin et al., 2009). At the time of our data collection in April, knowledge about the new virus was still limited and some experts (e.g., Kalager et al., 2020) claimed that in countries with a good healthcare system COVID-19 had a lower death toll than seasonal influenza and were skeptical of lockdowns and of dramatic presentations of the disease in the media. Participants at the time of the data collection could also have encountered such statements in traditional media including the leading serious newspaper in Norway “Aftenposten” (e.g., Berg Bentzrød and Dommerud, 2020) which is why in our study we do not categorize statements downplaying the severity of the COVID-19 threat as misinformation. However, such attitudes, particularly when mediated by traditional news sources, may be far more impactful both in terms of their credibility, as well as in terms of their effect on precautionary behavior, than irrationally sounding misinformation stories from social media platforms, which tend to be disregarded by most of the population. In future studies it would be interesting to investigate whether the level of variation in COVID-19 threat appraisals presented by experts and authorities in different countries has any association with population attitudes, precautionary behavior and the actual spread of the virus. Another interesting topic would be to explore how attitudes and behavior in relation to COVID-19 develop over time within the same country and their association with the local progress of the pandemic. Studying the topic of attitudes toward COVID-19 threat is also important for future pandemics as, when facing an infectious disease for which there is no medication, people's beliefs and related behaviors are key for combating the disease and saving lives.

## Data Availability Statement

The raw data supporting the conclusions of this article will be made available by the authors, without undue reservation.

## Ethics Statement

The study involving human participants was reviewed and approved by the Institutional Review Board of Simula Research Laboratory. The data collection for this study was conducted in accordance with the national legislation and the institutional requirements.

## Author Contributions

PF designed the study, analyzed the data, and wrote the manuscript. PA and JL provided discussions and feedback. KR conducted the advanced statistical analyses. All authors approved the final version of the manuscript.

## Conflict of Interest

The authors declare that the research was conducted in the absence of any commercial or financial relationships that could be construed as a potential conflict of interest.

## Footnotes

^1^COVID-19 Dashboard by the Center for Systems Science and Engineering (CSSE) at Johns Hopkins University (JHU). https://www.arcgis.com/apps/opsdashboard/index.html#/bda7594740fd40299423467b48e9ecf6.
