# Supplementary material for: What Should I Trust? Individual Differences in Attitudes to Conflicting Information and Misinformation on COVID-19
Source: Front Psychol. 2021 Jun 21;12:588478. doi: 10.3389/fpsyg.2021.588478 (PMC8262492; doi:10.3389/fpsyg.2021.588478)
Supplement: Supplementary file 1 [file Data_Sheet_1.PDF]

frontiers

Supplementary Material

Appendix

COVID-threat belief *(1 = fully disagree, 4 = fully agree)*

The COVID-19 pandemic is still at the start and many more people will die by the end of the year.
People who were infected with COVID-19 will experience long-term negative health impacts.
People can be infected with COVID-19 repeatedly.
Norway underestimates the risks of COVID-19 and has implemented too mild precautionary measures.
Norway is lifting the measures against COVID-19 too early.
Authorities claim that COVID-19 is under control in order to save the economy, even at the cost of lives.
COVID-19 is under control in Norway. (reversed)

COVID-threat skepticism *(1 = fully disagree, 4 = fully agree)*

Norway overreacted and the measures against COVID-19 were too strict.
Only Sweden has the rational approach to the pandemic.
The biggest risk of COVID-19 is creating a panic.
Norway is lifting the measures against COVID-19 too late.
Health measures introduced in relation to COVID-19 are a threat to democracy.
Only old and sick can die of COVID-19.
COVID-19 is not more dangerous than influenza.
The COVID-19 pandemic is almost over.

Misinformation on COVID-19 *(1 = fully disagree, 4 = fully agree)*

The magnitude of COVID-19 is exaggerated in order to persuade the world's population to take a vaccine.
COVID-19 first appeared in a country other than China.
The 5G network has an effect on the spread of COVID-19.
Consumption of the Corona brand of beer has an effect on the spread of COVID-19.
Mosquitoes transmit COVID-19.
Vaccine against COVID-19 will be available by summer.
Western countries are most affected by COVID-19 because they accepted most refugees.
Eating garlic prevents COVID-19 infection.

Trust to authorities regarding COVID-19 pandemic *(1 = fully disagree, 4 = fully agree)*

The Norwegian Institute of Public Health has handled the pandemic correctly.
Norway introduced the measures against COVID-19 early enough.
The World Health Organization has handled the pandemic correctly.

The official numbers of COVID-19 cases and deaths accurately represent the reality in Norway.
The authorities and the media are lying about COVID-19. (reversed)
The app “Smittestopp” will help to prevent the spread of COVID-19 in Norway.
Installation of the app “Smittestopp” is a safety risk. (reversed)

Health measures (0 = no, 1 = sometimes, 2 = yes)
I avoid using public transport.
I avoid visiting my family.
I avoid meeting friends.
I wash my hands much more often than before.
I avoid touching surfaces in public by bare hand (doorknobs, call buttons etc.)
I go to a grocery store much less often than before.
I use a face mask when I am in a public place.
I frequently disinfect surfaces such as phones, wallets etc.
I avoid touching my face when I am in a in public place.
I avoid shaking hands and other physical contact.
I keep larger distance from people.
